# Dutcher bodies in an IgA‐myeloma patient harbouring the *FGFR3*/*IGH* fusion

**DOI:** 10.1002/jha2.657

**Published:** 2023-02-17

**Authors:** Masahiro Manabe, Nobuhiro Sogabe, Satoru Nanno, Yuuji Hagiwara, Reiko Asada, Ki‐Ryang Koh

**Affiliations:** ^1^ Department of Hematology Osaka General Hospital of West Japan Railway Company Osaka Japan; ^2^ Department of Clinical Laboratory Osaka General Hospital of West Japan Railway Company Osaka Japan

**Keywords:** cytogenetics, morphology, multiple myeloma

1

A 73‐year‐old female was referred with anaemia. Laboratory tests showed a haemoglobin concentration of 8.0 g/dL, an IgA level of 6432 mg/dL, and a free light chain kappa/lambda ratio of 105.9. A bone marrow examination revealed a plasma cell frequency of 50.2% and a large number of Dutcher bodies (FIGURE 1, panel (A), *arrows*: ×100 objective, May‐Giemsa stain). Dutcher bodies varied in size, number, and location in the nucleus (FIGURE 1, panels (B)–(M), ×100 objective, May‐Giemsa stain). Although a chromosomal analysis showed a normal female karyotype, fluorescent in situ hybridization revealed *FGFR3*/*IGH* fusion signals in 72.7% of cells. Based on these findings, the patient was diagnosed with IgA‐kappa myeloma. Although the patient was treated with lenalidomide and dexamethasone followed by carfilzomib and dexamethasone, she died from myeloma 1 year and 1 month after being diagnosed.

**FIGURE 1:**
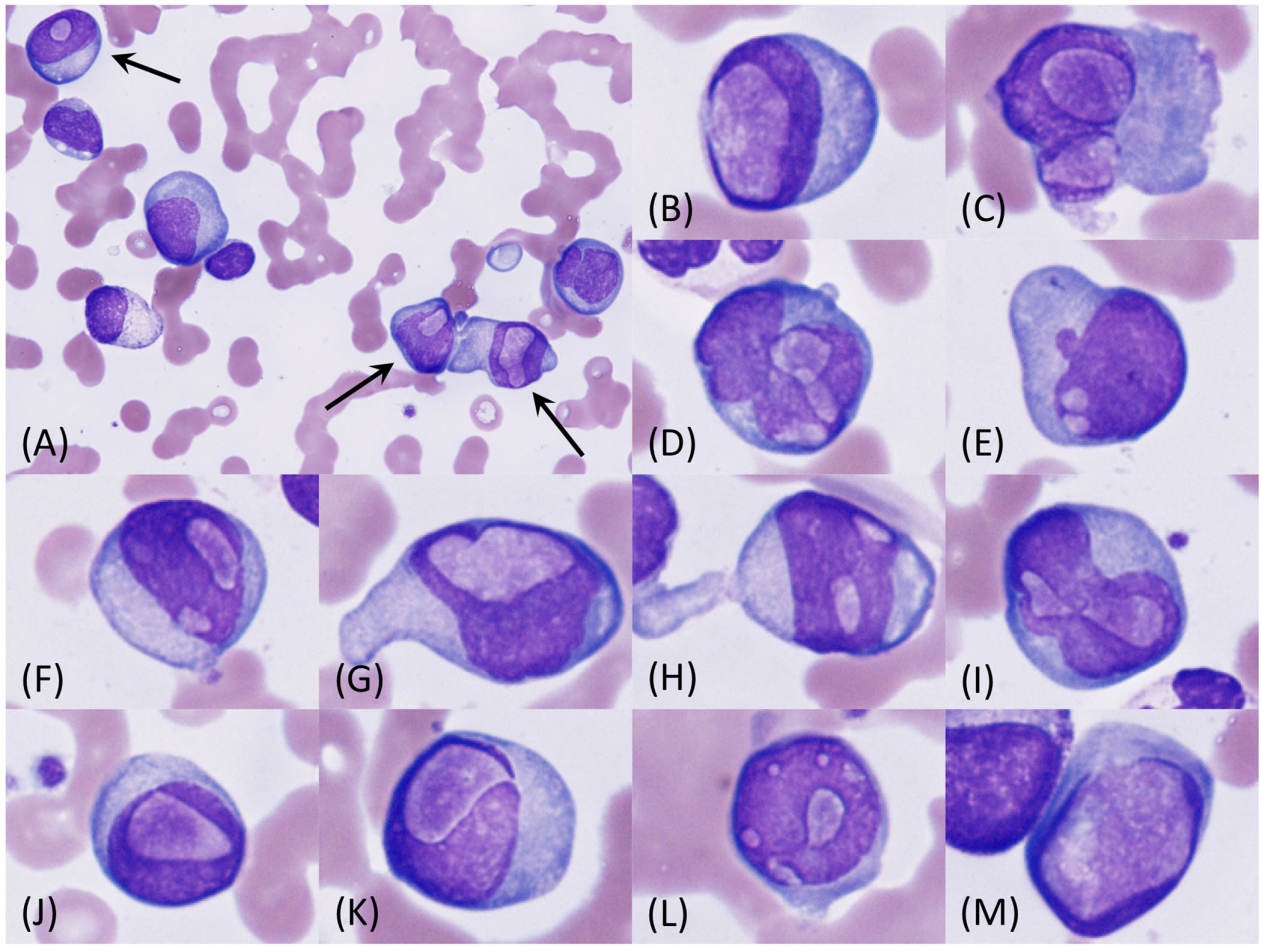
Bone marrow smear at the diagnosis of myeloma.

Dutcher bodies are closely associated with the translocation t(4;14), causing the *FGFR3*/*IGH* fusion, as well as the IgA isotype. The t(4;14) translocation is a poor prognostic factor for both overall survival and progression‐free survival; therefore, the t(4;14) translocation needs to be considered when Dutcher bodies are detected in myeloma patients.

## AUTHOR CONTRIBUTIONS STATEMENT

Masahiro Manabe, Nobuhiro Sogabe, Satoru Nannno, and Ki‐Ryang Koh designed the study. Masahiro Manabe, Yuuji Hagiwara, and Reiko Asada analyzed the data. Masahiro Manabe and Ki‐Ryang Koh collected the clinical data and specimens. Masahiro Manabe, Yuuji Hagiwara, and Reiko Asada wrote the manuscript. All of the authors have reviewed and approved the final manuscript.

## CONFLICT OF INTEREST STATEMENT

The authors have no competing interests to declare.

## FUNDING INFORMATION

There are no funding sources to declare.

## ETHICS STATEMENT

The patient provided her consent for the publication of this case report.

## Data Availability

All data generated during the present study are included in this manuscript.

